# Auxin-degron system identifies immediate mechanisms of OCT4

**DOI:** 10.1016/j.stemcr.2021.05.016

**Published:** 2021-06-17

**Authors:** Lawrence E. Bates, Mariana R.P. Alves, José C.R. Silva

**Affiliations:** 1 Wellcome-MRC Cambridge Stem Cell Institute, University of Cambridge, Cambridge CB2 0AW, UK; 2 Developmental Biology Unit, European Molecular Biology Laboratory, 69117 Heidelberg, Germany; 3 Department of Biochemistry, University of Cambridge, Cambridge CB2 1GA, UK

**Keywords:** Oct4, mouse nPSCs, *Pou5f1*, Nanog, embryonic stem cells, auxin-inducible degron

## Abstract

The pluripotency factor OCT4 is essential for the maintenance of naive pluripotent stem cells *in vitro* and *in vivo*. However, the specific role of OCT4 in this process remains unknown. Here, we developed a rapid protein-level OCT4 depletion system that demonstrates that the immediate downstream response to loss of OCT4 is reduced expression of key pluripotency factors. Our data show a requirement for OCT4 for the efficient transcription of several key pluripotency factors and suggest that expression of trophectoderm markers is a subsequent event. In addition, we find that NANOG is able to bind to the genome in the absence of OCT4, and this binding is in fact enhanced. Globally, however, the active enhancer-associated histone mark H3K27ac is depleted. Our work establishes that, while OCT4 is required for the maintenance of the naive transcription factor network, at a normal embryonic stem cell levels it antagonizes this network through inhibition of NANOG binding.

## Highlights

• OCT4 is required for enhancer activity and transcription of key pluripotency genes
• Exit from pluripotency precedes trophectoderm gene expression on loss of OCT4
• NANOG is able to bind to the genome in the absence of OCT4
• OCT4 reduces the capacity of NANOG to bind to the genome

## Graphical abstract

**Figure:**
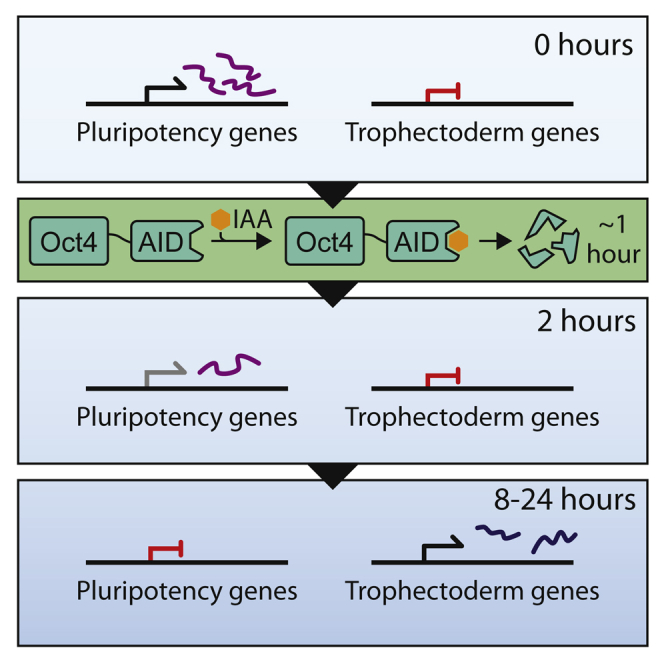

## Teaser

In this article, Silva and colleagues show that the initial response to the loss of OCT4 in mouse nPSCs is epigenetic and transcriptional silencing of key pluripotency genes, followed by the expression of trophectoderm-associated genes. Furthermore, they demonstrate that NANOG maintains its capacity to bind to the genome, and this is enhanced in the absence of OCT4.

## Introduction

Naive pluripotent stem cells (nPSCs) are the embryonic founders of the cells present in the adult animal. The transcription factor OCT4, expressed from the gene *Pou5f1*, is necessary for the maintenance of naive pluripotency *in vivo* and *in vitro*. In both cases, loss of OCT4 ultimately leads to the exit from naive pluripotency and cells taking on characteristics of the trophoblast lineage, including expression of the marker genes *Cdx2*, *Pl-1*, and *Eomes* and adoption of trophectoderm-like morphology (Nichols et al., 1998; Niwa et al., 2000).

Despite this, it is not understood why OCT4 is essential to the naive state; OCT4 is a highly promiscuous transcription factor, binding to thousands of sites in the genome (King and Klose, 2017; Simandi et al., 2016), and many of its target genes are known not to be essential for the maintenance of nPSCs (Hall et al., 2009; Matoba et al., 2006; Zwaka, 2012). At the same time, no target has been identified that can rescue OCT4-loss-driven differentiation through either overexpression or knockout (Hall et al., 2009; Matoba et al., 2006). It therefore seems likely that differentiation upon loss of OCT4 is the result of misregulation of a number of genes rather than of any single critical target.

Studying the effects of loss of OCT4 is further complicated by the fact that OCT4 appears to function in a level-dependent manner (Niwa et al., 2000). Mouse nPSCs engineered to express reduced levels of OCT4 are recalcitrant to differentiation and in some cases can be maintained under minimal culture conditions (Karwacki-Neisius et al., 2013; Radzisheuskaya et al., 2013), including basal serum-free media devoid of otherwise essential growth factors and inhibitors of differentiation pathways. Expression of pluripotency-associated genes may be enhanced in this state, and responses to signaling cues may be altered. In particular, it appears that transcript and protein levels of the core pluripotency factor Nanog are negatively correlated with OCT4 expression (Karwacki-Neisius et al., 2013).

NANOG is a homeodomain transcription factor that co-binds with OCT4 at many enhancers to promote expression of other pluripotency genes (Chen et al., 2008; Loh et al., 2006). While NANOG is not absolutely required for maintenance of the naive identity (Chambers et al., 2007), loss of NANOG results in widespread differentiation and reduced self-renewal capacity (Chambers et al., 2007; Ivanova et al., 2006; Mitsui et al., 2003). Interestingly, binding of NANOG to the genome appears to be enhanced in self-renewing nPSCs expressing low levels of OCT4, in line with the increased overall level of NANOG protein (Karwacki-Neisius et al., 2013; Radzisheuskaya et al., 2013), while during differentiation induced by loss of OCT4, NANOG binding is reduced (King and Klose, 2017). However, the immediate effect of the total loss of OCT4 protein on NANOG binding to the genome remains unknown.

Conventional methods for depleting OCT4, such as genetic ablation via Lox-Cre systems or transcriptional repression using Tet-OFF-regulated transgenes, rely on natural degradation to remove Oct4 mRNA and protein. A result of this is that OCT4 persists for a long time. Consequently, responses to depletion of OCT4 are typically examined on the order of hours to days following manipulation (Hall et al., 2009; Niwa et al., 2000), making it impossible to discern whether results are related to OCT4 or simply to cells undergoing differentiation. Further complicating such analysis, microarray data have revealed changes in the expression of thousands of genes at early time points after OCT4 suppression, even as the majority of OCT4 protein remains (Hall et al., 2009). This means that such variation may represent a response to a change in OCT4 expression to lower than wild-type levels, previously described to enhance self-renewal capacity in nPSCs (Karwacki-Neisius et al., 2013; Radzisheuskaya et al., 2013). Thus, the nature of the essential role of OCT4 for the self-renewal of nPSCs remains elusive.

To overcome these confounding factors, we generated an OCT4 fusion protein to a full-length auxin-inducible degron (AID). This allows us to induce rapid protein-level depletion of OCT4, making it possible for the first time to study the immediate molecular responses to loss of OCT4. These revealed an unprecedented impact on the transcriptional activity of pluripotency-associated transcription factor genes and addressed a long-standing question regarding the requirement of OCT4 for the binding of a key pluripotency factor, NANOG, to regulatory sequences.

## Results

### Auxin-degron-tagged OCT4 sustains nPSC self-renewal and permits rapid loss of OCT4

**Figure 1:**
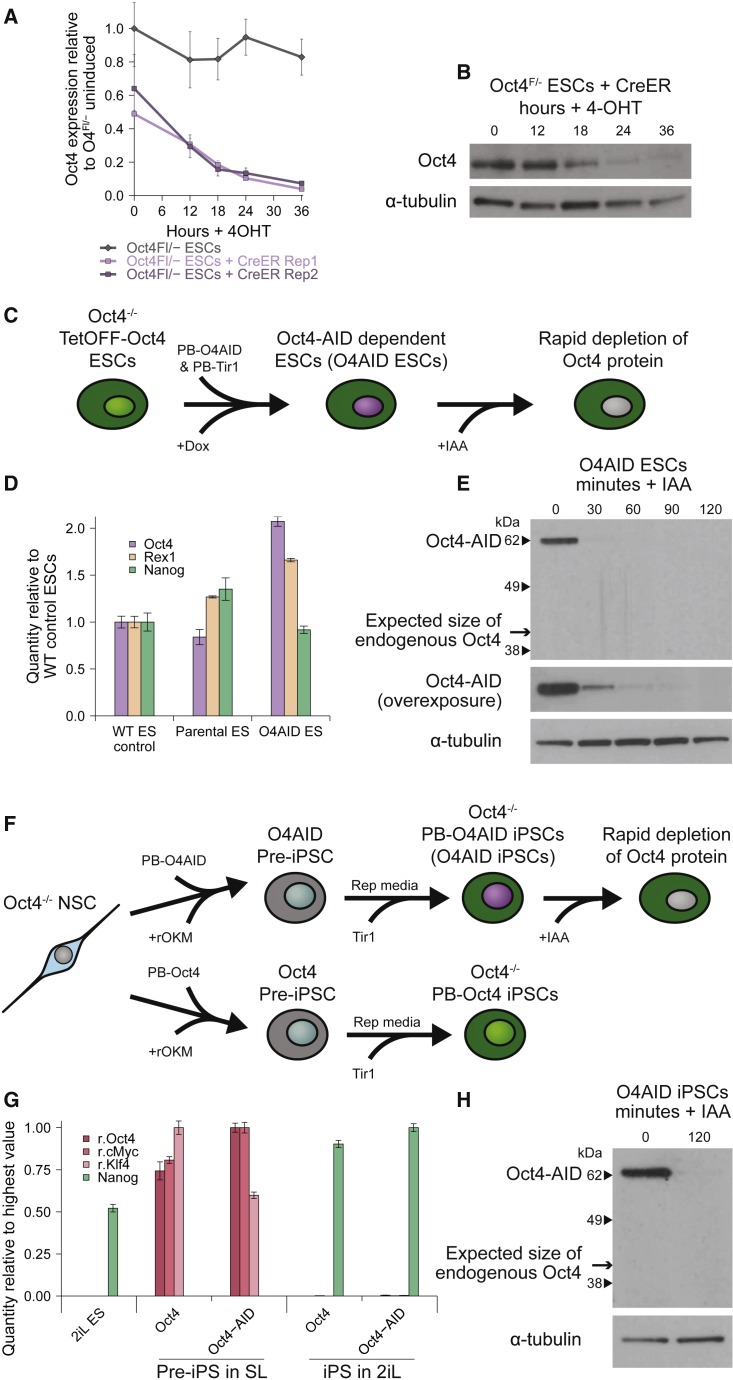
Auxin-degron-tagged OCT4 sustains nPSC self-renewal and permits rapid loss of OCT4 (A and B) The kinetics of OCT4 depletion in conventional Oct4^Fl/−^ ESCs were examined. (A) *Oct4* expression level (qRT-PCR) following addition of 4-OHT and medium change to SL. (B) OCT4 protein level (western blot) following addition of 4-OHT and medium change to SL. α-TUBULIN shown as a loading control. (C) Schematic showing the generation and use of O4AID ESCs. (D) Expression profiling (qRT-PCR) of pluripotency markers. (E) Oct4-AID fusion and wild-type OCT4 protein level (western blot) following addition of IAA. α-Tubulin was used as a loading control. (F) Schematic showing the generation and use of O4AID iPSCs. rOKM, retroviral Oct4, Klf4, and cMyc. (G) Expression profiling (qRT-PCR) of retroviruses and pluripotency factor *Nanog* in partially and fully reprogrammed cells. NSC, neural stem cell; r.Oct4, retroviral Oct4; r.cMyc, retroviral cMyc; r.Klf4, retroviral Klf4. (H) Oct4-AID fusion and wild-type protein level (western blot) following addition of IAA. qRT-PCR data represent the mean ± SD of three technical replicates. Dox, doxycycline; WT, wild type. See also Figure S1.

OCT4 protein has a relatively long half-life; unlike the pluripotency factor NANOG with a reported half-life of around 3 h, the half-life of OCT4 protein is typically found to be >6 h to as much as 24 h (Abranches et al., 2013; Muñoz Descalzo et al., 2013). In addition, the endogenous *Oct4* mRNA also appears to be unusually stable in mouse embryonic stem cells (ESCs) (Abranches et al., 2013). Indeed, we found that conventional tamoxifen-induced CreER-driven genetic ablation of *Pou5f1* resulted in a gradual reduction, and required over a day to fully deplete Oct4 RNA and protein (Figures 1A and 1B). Four days after induction, cells showed distinct morphological changes, resembling trophoblasts and more differentiated trophectodermal cells (Figure S1A). This shows that genetic and transcriptional methods of silencing Oct4 take a long time to fully deplete OCT4 protein, and cells are likely to experience a protracted period of low OCT4 content. Given the transcriptional and phenotypic effects previously observed in cells stably expressing low levels of OCT4 (Karwacki-Neisius et al., 2013; Radzisheuskaya et al., 2013), this is likely to have confounding effects on studies of immediate responses to complete removal of OCT4. We therefore decided to utilize a full-length AID tag (Baker et al., 2016; Nishimura et al., 2009) to achieve rapid, inducible protein-level degradation of OCT4 on addition of the small molecule indole-3-acetic acid (IAA).

Since OCT4 is essential for the maintenance of pluripotency, we sought to confirm that AID-tagged OCT4 retained its biological function by testing its capacity to support ESCs lacking wild-type OCT4. We utilized an existing Tet-OFF-Oct4 cell line; non-functional transgenes cannot rescue self-renewal from doxycycline-induced inhibition of wild-type OCT4 in these cells (Niwa et al., 2002), allowing them to be used in a complementation assay. We generated Oct4^−/−^ Tet-OFF-Oct4 Oct4-AID ESCs (O4AID ESCs hereafter) by transfecting these cells with constitutively expressed Piggybac Oct4-AID and NLS-Tir1 constructs and maintained the cells in the presence of doxycycline to inhibit expression of wild-type OCT4 (Figure 1C). Cells continued to proliferate and showed normal morphology (Figure S1B). Cells expressed the fusion protein with no detectable wild-type OCT4 present and did not show substantially altered expression of key pluripotency genes (Figures 1D and 1E). This demonstrates that the OCT4 fusion protein retains its original capacity to maintain naive pluripotency and is not substantially altered in its function by the addition of the AID domain.

To further validate the function of OCT4 within the fusion protein and to establish a second independent cell system, we generated induced PSCs (iPSCs) null for endogenous OCT4 and therefore wholly reliant on either transgenic wild-type OCT4 or OCT4-AID for their maintenance (Figure 1F). After a period of outgrowth, the cells initiated reprogramming and generated intermediate pre-iPSCs (Silva et al., 2008; Theunissen et al., 2011) with high expression of retroviral reprogramming factors but very low expression of the pluripotency factor *Nanog* (Figure 1G). Under naive-specific conditions, colonies with a domed, iPSC-like morphology were readily obtained using either wild-type or Oct4-AID constructs (Figure S1C). *Nanog* was robustly expressed, while retroviral transgenes were efficiently silenced in the fully reprogrammed iPSCs (Figure 1G). Again, as expected, wild-type OCT4 was not detectable, while the OCT4-AID fusion protein was robustly expressed (Figure 1H). Furthermore, under differentiation conditions, the cells showed loss of pluripotency markers and upregulation of markers of all three germ layers, indicating competency to initiate normal differentiation (Figure S1D). The ability of the Oct4-AID transgene to maintain pluripotency in this OCT4 knockout background further demonstrates that the essential function of OCT4 is maintained.

Utilizing the new protein degradation system, tagged OCT4 protein levels could be greatly reduced within half an hour and undetectable within 2 h of addition of IAA (Figures 1E and 1H). This quick turnover means that there is no appreciable OCT4-low state, and we therefore sought to use this system to study immediate responses of cells to total loss of OCT4.

### OCT4 is required for the expression of key pluripotency factors

**Figure 2:**
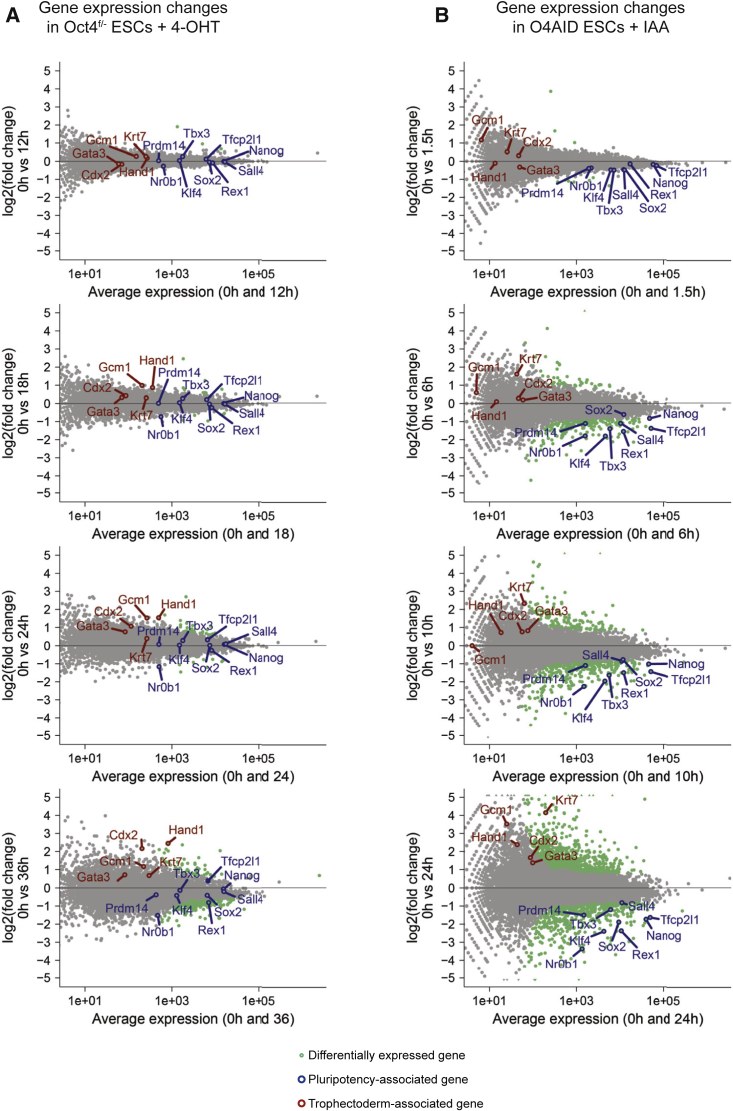
OCT4 is required for the expression of key pluripotency factors (A and B) MA plots showing gene expression (RNA-sequencing) changes following (A) deletion of *Pou5f1* in conventional Oct4^F/−^ CreER ESCs by addition of 4-OHT or (B) degradation of OCT4 protein in O4AID ESCs by addition of IAA. Differentially expressed genes are highlighted in green (q > 0.9, NOISeq-sim). Selected pluripotency- (blue) and differentiation- (red) associated genes are indicated. (A) Top to bottom: 0 versus 12, 0 versus 18, 0 versus 24, and 0 versus 36 h. (B) Top to bottom: 0 versus 1.5, 0 versus 6, 0 versus 10, and 0 versus 24 h. Note that for Oct4^F/−^ CreER ESCs two replicate datasets were merged, and for O4AID ESCs a single replicate was used. A non-parametric algorithm designed for use on data lacking replicates was used for the analysis (see experimental procedures for more details). Key findings were corroborated by qRT-PCR analysis in two independent cell lines; see Figure S2.

We analyzed gene expression changes following conventional tamoxifen-induced CreER-driven genetic ablation of *Pou5f1* (Figures 2A and S2A). The results indicate that, while specific pluripotency-associated genes such as *Nr0b1* are downregulated within 24 h of induction of OCT4 depletion, upregulation of trophoblast-associated genes occurs concurrent with expression of most naive pluripotency factors; overall, the pluripotency network remains expressed for 24 h. In keeping with previous observations, however, many of these gene expression changes are detected while significant amounts of OCT4 protein are still present (Figure 1B). This is consistent with reports that expression of pluripotency genes may be enhanced in cells with reduced levels of OCT4 (Karwacki-Neisius et al., 2013; Radzisheuskaya et al., 2013), supporting the notion that conventional methods of OCT4 depletion pass through a protracted OCT4-low state, complicating interpretation of the effects of removal of OCT4.

In contrast to the above, analysis of O4AID cells indicated that expression of naive-associated factors is quickly extinguished on loss of OCT4 (Figures 2B, S2C, and S2D). Factors such as *Tfcp2l1*, *Klf4*, and *Tbx3* are rapidly downregulated following targeted OCT4 protein depletion, whereas they were actively maintained in the slower conventional system.

Examining several trophoblast-associated genes confirmed that these cells exit pluripotency following loss of OCT4 and differentiate toward trophectodermal lineages (Figures 2B, S2C, and S2D), in keeping with conventional OCT4-depletion systems. However, strong upregulation of trophectoderm-associated genes is seen only after a decrease in pluripotency marker expression using this system, indicating that the decision to enter this extraembryonic identity is made only after cells have begun to exit the naive state. Analysis of eGFP-AID ESCs validated that neither loss of pluripotency gene expression nor upregulation of trophectoderm markers was caused by IAA itself, nor activation of TIR1-induced proteasomal degradation (Figure S2B).

Together these altered kinetics in transcriptional responses highlight that there are important differences between gradually reducing OCT4 protein levels and removing OCT4 protein entirely.

### Enhancers show rapid epigenetic and functional inactivation following loss of OCT4

**Figure 3:**
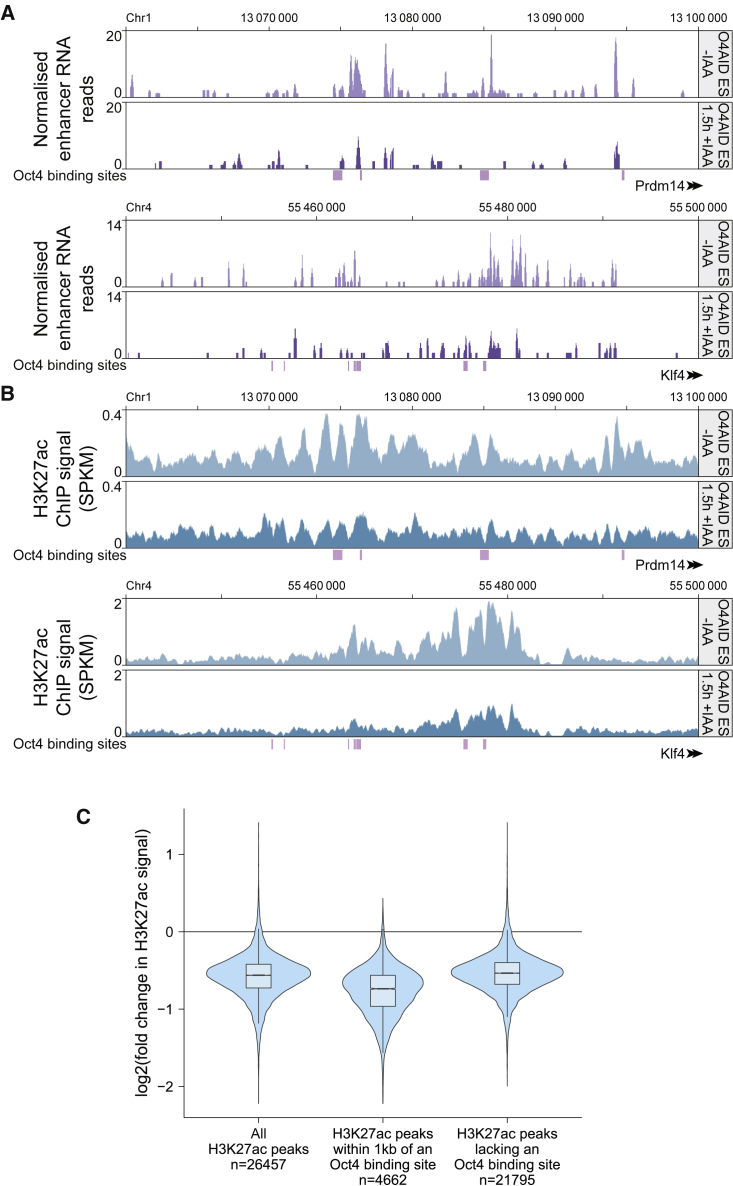
OCT4 is required for enhancer activity at key pluripotency loci and for maintaining global H3K27ac (A) Mapped RNA-sequencing reads (single replicate) of enhancer RNAs at the *Prdm14* distal enhancer (top) and the *Klf4* distal enhancer (bottom) in O4AID ESCs before and 1.5 h after addition of IAA. (B) Visualization of H3K27ac ChIP-seq signal (signal per kilobase per million mapped reads) at the *Prdm14* distal enhancer (top) and the *Klf4* distal enhancer (bottom) in O4AID ESCs before and 1.5 h after addition of IAA. Genomic coordinates refer to the GRCm38/mm10 genome assembly, and gene intron/exon annotations are taken from Ensembl. OCT4 binding sites generated from ChIP-seq data from Marson et al. (2008) are indicated in purple. (C) Violin and box plot showing log2-fold change in H3K27ac signal between uninduced and 1.5 h IAA-treated O4AID ESCs. Data were generated by merging mapped ChIP-seq reads from three independent immunoprecipitations. Note that the key findings were corroborated by ChIP-qPCR in two independent cell lines (Figure S3B). Boxes show the median value and extend to the 25th and 75th quartiles, and whiskers extend to 1.5 times the interquartile range. All H3K27ac peaks (n = 26,457) above a (background) threshold and the complementary subsets of peaks within 1 kb of an OCT4 binding site (n = 4,662) and not within 1 kb of an OCT4 binding site (n = 21,795) are plotted. For each set a paired t test of H3K27ac signal before and after treatment showed highly significant change, p < 10^−10^. See also Figure S3.

Given the rapid downregulation of pluripotency-associated genes, we examined how enhancer elements were affected by loss of OCT4 at a number of these loci. Enhancer-associated transcription can be observed at many active enhancers (De Santa et al., 2010; Kim et al., 2010), and can play a functional role in promoting transcription of target genes (Alvarez-Dominguez et al., 2017; Shii et al., 2017). We observed loss of transcription from several pluripotency enhancer elements shortly after ablation of OCT4 (Figure 3A), implying loss of enhancer activity.

To validate this, we looked at the level of acetylation of histone H3 at lysine residue 27 (H3K27ac), a mark closely associated with active enhancers. Using chromatin immunoprecipitation sequencing (ChIP-seq), we observed a dramatic decrease in H3K27ac at pluripotency-associated enhancers (Figures 3B and S3A), and we confirmed this using ChIP-qPCR in both O4AID ESC and O4AID iPSC systems (Figures S3B and S3C). Again, we examined eGFP-AID ESCs, and found no change in H3K27ac levels at pluripotency-associated enhancers following application of IAA, demonstrating that this response is specifically a response to OCT4 depletion (Figures S3B and S3C). Loss of H3K27ac was not restricted to pluripotency-associated loci, however; global analysis of all H3K27ac peaks showed a significant decrease (Figure 3C). Using peaks called from OCT4 ChIP-seq data generated by Marson et al. (2008), we examined if this was the result of decommissioning of OCT4-co-bound enhancers; while the reduction in H3K27ac signal was stronger at sites close to OCT4 binding sites, H3K27ac peaks that were not directly bound by OCT4 still showed a marked reduction, indicating a remarkable shift in the global chromatin landscape in the absence of OCT4.

### OCT4 is dispensable for NANOG binding to pluripotent regulatory sequences

**Figure 4:**
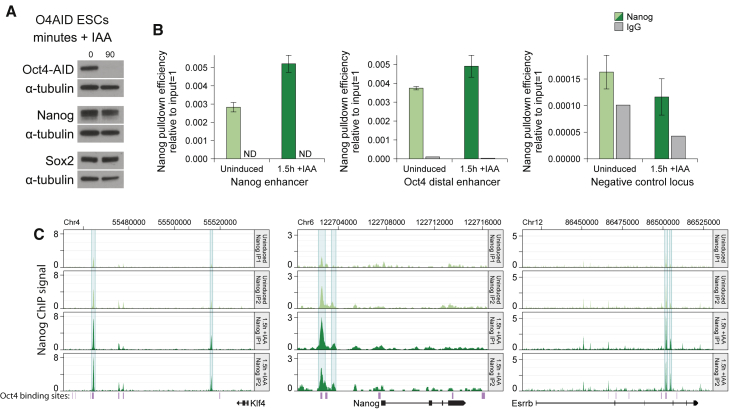
OCT4 is dispensable for NANOG binding to pluripotent regulatory sequences (A) Protein level of OCT4 and NANOG (western blot) in O4AID ESCs before and 1.5 h after addition of IAA, with α-tubulin as a loading control. (B) ChIP qPCR following pull-down of NANOG or using normal immunoglobulin G (IgG) negative control at NANOG binding sites or a negative control locus in O4AID ESCs. ChIP qPCR data for NANOG pull-down represent the mean ± SD of three IPs; IgG pull-down represents the mean of three technical replicates of a single IP. ND, not detected. Note that this is corroborated by ChIP qPCR in an independent cell line (Figure S4). (C) Visualization of NANOG ChIP-seq signal (signal per kilobase per million mapped reads) across indicated loci before and 1.5 h after addition of IAA, shown for two IPs. Genomic coordinates refer to the GRCm38/mm10 genome assembly, and gene intron/exon annotations are taken from Ensembl. OCT4 binding sites generated from ChIP-seq data from Marson et al. (2008) are indicated in purple. See also Figure S4.

Depletion of OCT4 was so rapid that protein levels of other pluripotency factors were not strongly affected by the time the OCT4 protein was fully removed; NANOG protein levels were only slightly reduced (Figures 4A and S4C). As a result, we decided to use this opportunity to examine changes in the chromatin binding profile of NANOG, a transcription factor that binds to many of the same enhancer elements as OCT4.

NANOG binding efficiency at the enhancer of the *Nanog* and *Pou5f1* genes was analyzed by ChIP-qPCR. Surprisingly, in both O4AID ESC and iPSC systems, NANOG not only remained bound, but there was greater NANOG signal following depletion of OCT4 (Figures 4B and S4D). Interestingly, other work has suggested that in the OCT4-low state NANOG has increased genomic occupancy, although OCT4 was still present (Karwacki-Neisius et al., 2013; Radzisheuskaya et al., 2013). We validated that Oct4AID was effectively depleted by immunoprecipitating OCT4 from sheared chromatin, followed by western blotting. While we could readily pull down Oct4AID from uninduced samples, it was almost undetectable, even in the chromatin-enriched eluate in IAA-treated samples (Figure S4A). In the data presented here, we actively avoid the presence of OCT4, suggesting that, either directly or indirectly, OCT4 actively reduces the ability of NANOG to bind to enhancer elements.

We therefore examined the NANOG binding profile at the regulatory regions of several key pluripotency genes using ChIP-seq. There was a clear increase in NANOG binding at several loci following depletion of OCT4 (Figure 4C). Interestingly, expression of these genes was decreased following induced OCT4 degradation despite the increased NANOG binding (Figures 2B, S2C, and S2D). Of particular note, the *Klf4* distal enhancer element showed greater NANOG signal following loss of OCT4, yet reduced mRNA and enhancer RNA (eRNA) expression, and H3K27 acetylation (Figures 2B, S2C, S2D, 3A, 3B, S3B, and S3C). This further highlights that the presence of OCT4 appears to be absolutely required for the expression of certain pluripotency-associated genes.

### NANOG binding is increased globally, independent of OCT4 co-binding

**Figure 5:**
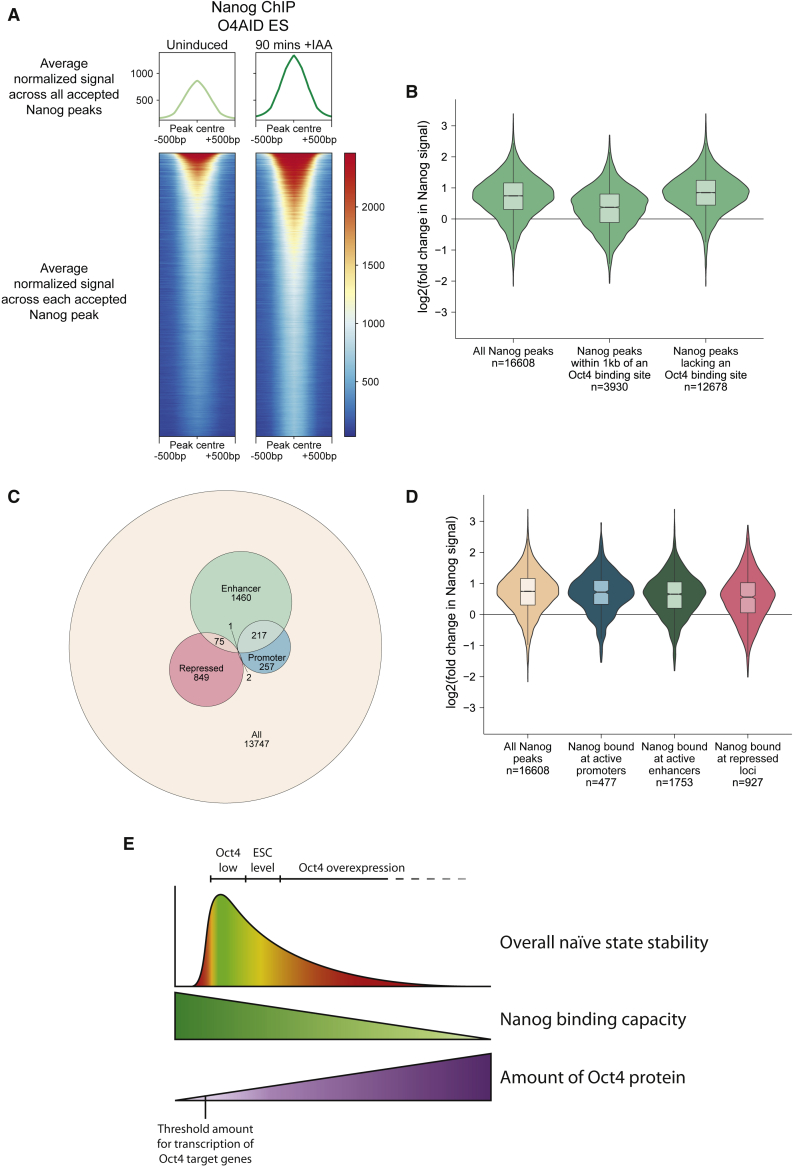
NANOG binding is increased globally, independent of OCT4 co-binding (A) Summary distribution (top) and heatmap (bottom) of NANOG signal (sum of mapped reads from two independent IPs, normalized to library size) centered at the summit of all NANOG binding sites across the genome, before and 1.5 h after addition of IAA. (B) Violin and box plot of log2 of the fold change in the average normalized NANOG signal at each NANOG peak in the genome (n = 16,608) before and after addition of IAA, further broken down into loci within 1 kb of (n = 3,930) or farther away than 1 kb from (n = 12,678) the OCT4 binding sites generated from ChIP-seq data from Marson et al. (2008). Mapped reads from two independent IPs each, before and after treatment, were merged to generate NANOG ChIP-seq data. Boxes show the median value and extend to the 25th and 75th quartiles, and whiskers extend to 1.5 times the interquartile range. (C) Euler plot showing the number of NANOG peaks assigned to various chromatin environments and the overlap between assignations. Numbers indicate the number of peaks uniquely in that section of the diagram. (D) Violin and box plot of log2 of the fold change in the average normalized NANOG signal (data as in [B]) at each NANOG peak in the genome before and 1.5 h after addition of IAA, further broken down into non-exclusive chromatin environments, as indicated in (C). Boxes show the median value and extend to the 25th and 75th quartiles, and whiskers extend to 1.5 times the interquartile range. (E) Model indicating the proposed relationship between the quantity of OCT4 protein and the capacity for NANOG to bind to the genome and consequently the ability of cells to maintain a naive identity. See also Figure S5.

We further analyzed our NANOG ChIP-seq data to examine global changes in NANOG binding immediately following OCT4 ablation. Looking at all NANOG binding sites revealed a global shift toward greater levels of NANOG enrichment (Figures 5A and 5B). Examining the change in NANOG signal over all peaks, there is a significant overall increase following loss of Oct4 (16,608 NANOG peaks; paired t test of NANOG signal before and after treatment, p < 10^−10^; t test of log fold change of thresholded peaks versus 4,458 sub-threshold [background] peaks, p < 10^−10^). To investigate whether the effect on NANOG binding was specific to sites where OCT4 co-binds, we looked at the complementary subsets of NANOG peaks less than 1 kb from an OCT4 peak (3,930 sites) and those that do not co-bind with OCT4 (12,678 sites), again using peaks called from OCT4 ChIP-seq data from Marson et al. (2008) (Figure 5B). At OCT4-NANOG co-binding sites there was a detectable increase in NANOG signal; globally this was statistically significant (paired t test of NANOG signal before and after treatment, p < 10^−10^; t test of log fold change of thresholded peaks versus 382 sub-threshold [background] peaks, p < 10^−10^), although surprisingly, the magnitude of the increase was reduced compared with all NANOG peaks. NANOG peaks that do not overlap with OCT4 displayed a more dramatic increase in NANOG binding (12,678 peaks; paired t test of NANOG signal before and after treatment, p < 10^−10^; t test of log fold change of thresholded peaks versus 4,076 sub-threshold [background] peaks, p < 10^−10^), suggesting that the reduced NANOG binding in the presence of OCT4 is not directly due to physical occlusion of NANOG binding sites by OCT4 or due to changes in local chromatin structure in the presence or absence of OCT4. One possible explanation for this global effect is an increase in the stability of NANOG protein following loss of OCT4; however, it has previously been shown that NANOG displays increased stability in the presence of OCT4 (Muñoz Descalzo et al., 2013). In keeping with this, we determined the half-life of NANOG with or without addition of IAA, which revealed a decrease from ∼2.3 h in the presence of OCT4 to ∼1.4 h in the absence of OCT4 (Figure S5A). This suggests that stability is not the cause of the increased genomic occupancy of NANOG that we observed.

We additionally examined the effect of chromatin context on the change in NANOG binding following loss of OCT4. We used publicly available ChIP-seq data to classify 2,861 NANOG peaks as enhancer, promoter, and/or repressive mark associated. As expected, the majority of these NANOG peaks were associated with enhancers (Figure 5C). In all cases, there was a significant increase in NANOG signal (Figure 5D; paired t test, p < 10^−10^) and the mean increase in signal was comparable, suggesting that the chromatin context has little impact on the ability of OCT4 to restrict NANOG binding under normal conditions.

## Discussion

It has been shown that OCT4 is dispensable for the initial generation of the blastocyst structure, but required for segregation of the inner cell mass into naive epiblast and primitive endoderm (Le Bin et al., 2014). OCT4 is also essential for maintaining naive pluripotency *in vitro* (Niwa et al., 2000). Despite this, the manner in which OCT4 is required for these processes is unclear; no overexpression or knockout mutants that rescue the OCT4 knockout phenotype have been identified (Hall et al., 2009; Matoba et al., 2006; Niwa et al., 2000). To infer how OCT4 mediates self-renewal, we wanted to examine initial transcriptional changes in response to OCT4 depletion. It is notable that similar experiments have been performed before (Hall et al., 2009; Matoba et al., 2006) and have failed to yield an essential role for OCT4. However, it is known that cells exhibiting a low level of OCT4 can sustain expression of pluripotency markers under mild differentiation conditions (Karwacki-Neisius et al., 2013; Radzisheuskaya et al., 2013), and we wondered if transcription of such factors was being artificially maintained by the gradually reducing level of OCT4 protein in conventional depletion systems. The half-life of OCT4 is sometimes reported to be very long (Lin et al., 2012; Wei et al., 2007); it took a full day for OCT4 to be fully removed from *Pou5f1*^F/−^ cells used in this work (Figures 1A and 1B), and published work shows more than 10 h for complete OCT4 depletion using a Tet-OFF system (Hall et al., 2009; Niwa et al., 2000). This is a significant amount of time, especially since transcriptional changes are already observed within this window. To avoid this confounding factor, we established new cell lines utilizing rapid depletion of OCT4 at the protein level (Figures 1C–1H). It has previously been reported that the AID can reduce the half-life of tagged proteins to ∼20 min in mammalian systems (Holland et al., 2012; Nishimura et al., 2009), and indeed we found that tagged OCT4 protein was greatly reduced within half an hour and fully depleted within 1.5–2 h (Figures 1E and 1H). After ensuring that both the AID domain and OCT4 were unaltered in their function in this fusion protein (Figures 1D, 1G, S1B, and S1C), we proceeded to reexamine the immediate effects of loss of OCT4. Since depletion of OCT4 was so rapid, we were able to examine the effects of loss of OCT4 prior to extensive changes in the protein level of other pluripotency transcription factors. Crucially, the high temporal resolution afforded by such a rapid depletion system reveals two phases of transcriptional change following loss of OCT4, unlike following genetic ablation (Figure 2). First, RNA levels of all pluripotency factors examined were rapidly decreased following degradation of OCT4 protein. Notably, all the factors tested display OCT4 binding at their enhancer or promoter elements in published ChIP-seq data. This suggests that the presence of OCT4 at these elements may be essential for the expression of a broad range of pluripotency-associated genes, explaining why no single factor can rescue the OCT4 knockout phenotype. Only subsequently did we observe upregulation of trophectoderm-associated markers, suggesting that this may be a secondary effect or that derepression of these loci occurs over a longer timescale. In keeping with an essential role in permitting active transcription, we found a global reduction in the level of H3K27ac, associated with active enhancers (Figures 3 and S3). The fact that this loss of active enhancer marks extends beyond pluripotency-associated loci could imply that OCT4 is critical for maintaining the uniquely permissive chromatin environment found in naive pluripotent cells, beyond simply acting to drive expression of key pluripotency factors.

ChIP-qPCR and ChIP-seq against NANOG protein yielded an interesting and surprising result; in the absence of OCT4, greater levels of NANOG were found bound to the genome (Figures 4, 5, S4,and S5). Remarkably, this extended beyond OCT4 co-bound sites, with an increase in NANOG levels observed at many binding sites lacking OCT4 (Figure 5B). Consequently, it seems unlikely that this effect is due to OCT competing with NANOG for binding sites, in keeping with previous reports of simultaneous binding of OCT4 and NANOG to regulatory regions as detected by sequential ChIP (Medeiros et al., 2009). A previous study examining the localization of NANOG and SOX2 24 h after transcriptional depletion of OCT4 found that SOX2 and NANOG binding was reduced at many OCT4 co-bound sites following silencing of *Oct4* (King and Klose, 2017). However, this carries the caveat that such changes may be due to subsequent transcriptional and epigenetic changes downstream of the loss of OCT4 rather than direct effects. In particular, reduced NANOG signal was associated with loss of transcription and chromatin accessibility, suggesting that these enhancer elements were no longer active or accessible, perhaps due to the onset of differentiation, by the time of their analysis. In contrast, by using a targeted protein degradation system, we are able to study the immediate changes in protein localization in our system, limiting secondary effects.

Interestingly, we observed a rapid downregulation of *Klf4*, an eRNA associated with the *Klf4* super-enhancer, and loss of the active enhancer histone mark H3K27ac at this locus, despite an increase in NANOG binding in this region. It has previously been shown that expression of *Klf4* is highly dependent on the presence of NANOG, although it can be partially rescued with overexpression of ESRRB (Festuccia et al., 2012). However, only when NANOG and STAT3 are both present is there efficient transcription of both *Klf4* and the *Klf4* eRNAs; neither overexpression of NANOG in the absence of active STAT3 nor induction of JAK/STAT signaling in NANOG knockout ESCs is capable of inducing *Klf4* or *Klf4* eRNA upregulation (Stuart et al., 2014). Our findings appear to add OCT4 to this list of factors that need to be present to achieve activation of this enhancer region.

It is currently unclear how the global nuclear structure changes following acute depletion of OCT4. Previous work has suggested that OCT4 may act as a pioneer factor, opening chromatin that would otherwise be highly compacted (King and Klose, 2017; Soufi et al., 2015). As a result, removal of OCT4 might result in the eviction of other transcription factors and repression of gene expression, although the increased binding of NANOG to chromatin suggests this may not be the case (Figures 4 and 5). In addition, there could be significant changes in the chromatin architecture due to altered topological domains in the absence of OCT4. Decommissioning of super-enhancers, many of which are bound by OCT4 in ESCs (Whyte et al., 2013), could lead to a reorganization of the nuclear structure, as these elements interact with multiple promoter regions, even over large distances (Novo et al., 2018).

The ability to probe transcriptional responses immediately following total OCT4 depletion, and the capacity to examine changes in the binding of NANOG to chromatin prior to significant secondary effects, has allowed us to go some way in explaining past discrepancies in the perceived role of OCT4 in the pluripotency network. As Hall et al. note, *Nanog* has been described as an OCT4 target gene, and there is significant biochemical data validating that OCT4 binds to the *Nanog* enhancer region and is required for its expression (Kuroda et al., 2005; Rodda et al., 2005), yet gradual OCT4 depletion appeared to have a limited, even positive impact on *Nanog* mRNA (Figures 2A and S2) (Hall et al., 2009). We propose that the reason for this is that, while OCT4 may be essential for *Nanog* expression, above a minimal level OCT4 negatively regulates the binding of other transcriptional activators. Thus, as OCT4 levels drop and cells pass through an OCT4-low state, binding of OCT4 to the *Nanog* enhancer is reduced and other transcription factors can be recruited, buffering *Nanog* expression levels. Once OCT4 is entirely depleted *Nanog* expression falls, as the locus is no longer able to be transcribed.

In agreement with existing literature, it appears that the half-life of NANOG is increased in the presence of OCT4 (Figure S5A) (Muñoz Descalzo et al., 2013). As a result, increased NANOG stability is not likely to be responsible for the increased genomic occupancy we observed. However, it would be interesting to see what other properties of NANOG protein are altered in the absence of OCT4, particularly whether changes in the NANOG protein interactome or DNA binding affinity could explain the increase in chromatin binding. Differences in post-translational modifications could affect the ability of NANOG to interact with DNA and proteins in this way.

Combining the transcriptional responses to loss of OCT4 with the observed global changes in NANOG ChIP signals has led us to a unifying model for the range of phenotypes associated with different levels of OCT4 protein in ESCs (Figure 5E). In the absence of OCT4 key pluripotency factors cannot be expressed and cells cannot maintain a naive identity. With low levels of OCT4, a threshold is reached such that these factors are able to be expressed, and efficient binding by NANOG results in robust transcription of the whole naive network to such an extent that differentiation is compromised. At higher OCT4 levels, such as seen in wild-type ESCs and embryos, OCT4 globally suppresses the binding of NANOG, resulting in a weaker transcriptional network. This allows cells to undergo differentiation in response to signaling cues. On overexpression of OCT4, it is expected that the binding of NANOG to chromatin will be further reduced, destabilizing the naive network to the point that it cannot be readily maintained.

In summary, utilizing a rapid protein-level-depletion strategy, we identified the primary transcriptional response to loss of OCT4 as a decrease in the expression of pluripotency-associated genes, and upregulation of trophectoderm factors is a subsequent event. In addition, we found a global increase in the amount of NANOG associated with the genome in the absence of OCT4, suggesting a mechanism by which wild-type levels of OCT4 ensure that naive cells retain the capacity to initiate differentiation in response to appropriate signals. Together, these reveal a model that ties together the range of phenotypes associated with differing levels of OCT4, concisely explaining how different levels of this factor result in seemingly contradictory cell behaviors.

## Experimental procedures

### Cell culture

Mouse ESCs were cultured under 2iL conditions as previously described. Briefly, cells were maintained in N2B27 (1:1 DMEM/F-12 and Neurobasal, 2 mM L-glutamine, 1× penicillin-streptomycin, 0.1 mM 2-mercaptoethanol, 1% B27, 0.5% N2) supplemented with 3 μM CHIR99021, 1 μM PD0325901, and 20 ng/mL mouse leukemia inhibitory factor (mLIF). Where stated, cells were transitioned into SL conditions, consisting of GMEM without L-glutamine, 10% fetal bovine serum, 1× non-essential amino acids, 1 mM sodium pyruvate, 2 mM L-glutamine, 1× penicillin-streptomycin, 0.1 mM 2-mercaptoethanol, and 20 ng/mL mLIF. ESCs and iPSCs were maintained on gelatin-coated tissue culture plastic. Neural stem cells (NSCs) were maintained on laminin-coated tissue culture plastic in N2B27 supplemented with 10ng/ml EGF and 20ng/ml FGF2. Cells were passaged every 2–4 days using Accutase as required. Where described, the medium was supplemented with 500 nM 4-OHT, 1 μg/mL doxycycline, and/or 500 μM IAA.

### Cell lines

Oct4^F/−^ ESCs (Oct4^F/β-geo^) were previously derived from a cross between Oct4^+/β-geo^ and Oct4^F/F^ mice. O4AID ESCs were generated by transfecting ZHBTc4 ESCs (Niwa et al., 2000) with pPB-CAG-Oct4AID-PGK-Hph (mouse Oct4 tagged at the C terminus with full-length AID, separated by a short 2 amino-acid linker consisting of Proline-Glycine) and pPB-CAG-Tir1-IRES-Bsd (*Oryza sativa* Tir1, with an N-terminal SV40 nuclear localization sequence signal, codon optimized for expression in mouse), with pPBase to achieve efficient integration, and maintained in the presence of doxycycline to silence the Tet-OFF-Oct4 transgene. O4AID iPSCs were generated from Oct4^−/−^ NSCs (Radzisheuskaya et al., 2013) nucleofected with pPB-CAG-Oct4AID-PGK-Hph and pPBase to achieve efficient integration and reprogrammed; equivalent wild-type Oct4 control cells were generated by nucleofection with pPB-CAG-Oct4-PGK-Hph and pPBase. The generated O4AID iPSCs were transfected with pCAG-Tir1-IRES-Bsd, and clonal lines were assessed for functional transgene integration. eGFP-AID ESCs were generated by transfecting Oct4^F/−^ ESCs with pPB-CAG-eGFPAID-IRES-Zeo (eGFP tagged at the C terminus with full-length AID, separated by a short PG linker) and pPB-CAG-Tir1-IRES-Bsd with pPBase.

### Reprogramming

PLAT-E cells were transfected with pMXs-Oct4, pMXs-Klf4, or pMXs-cMyc using FuGENE 6 reagent to produce retroviral particles. The medium was changed the following day, and after 48 h supernatant containing retroviral particles was collected. The media were filtered and mixed together in an equal ratio, then Polybrene was added to a final concentration of 4 μg/mL. The Polybrene/virus mixture was applied to NSCs. Twenty-four hours later, NSCs were nucleofected with a 1:5 ratio of pPBase and either pPB-CAG-Oct4AID-PGK-Hph or pPB-CAG-Oct4-PGK-Hph using Amaxa Nucleofector technology and plated in NSC medium for 2 days; then they were switched to SL medium. The medium was then switched to KSR-2iL (GMEM without L-glutamine, 10% KOSR, 1% fetal bovine serum, 1× non-essential amino acids, 1 mM sodium pyruvate, 2 mM L-glutamine, 1× penicillin-streptomycin, 0.1 mM 2-mercaptoethanol, and 20 ng/mL mLIF). Selection was applied for expression from the endogenous Oct4 locus on the ninth day in KSR-2iL. Once colonies had expanded they were passaged into 2iL conditions.

### Conventional differentiation

iPSCs were induced to differentiate either in monolayer differentiation (N2B27) or in suspension (embryoid body). For monolayer differentiation, 130,000 cells were plated in a gelatin-coated well of a six-well plate in 2iL medium. The following day this medium was removed; the wells were washed with N2B27 lacking CHIR99021, PD0325901, and LIF; and the cells were cultured in N2B27 alone for the duration of the time course. For suspension differentiation, 570,000 cells were transferred to a low-attachment 6 cm dish in serum-based medium lacking LIF. Cells were allowed to aggregate and expand in suspension for the duration of the time course. The medium was changed by gently centrifuging floating aggregates and transferred back to the low-attachment dish.

### qRT-PCR and RNA sequencing

Total RNA was isolated from cultured cells using the RNeasy Mini Kit (Qiagen) according to the manufacturer's instructions, including on-column DNase I digest. For qRT-PCR, RNA was reverse transcribed using SuperScript III First-Strand Synthesis SuperMix for qRT-PCR, and reactions were performed on a StepOnePlus real-time PCR system with recommended settings using the TaqMan Fast Universal PCR Mix or Fast SYBR Green Master Mix. See the supplemental experimental procedures for primers and TaqMan probes.

For high-throughput RNA sequencing, RNA integrity was assessed on a Qubit fluorometer (Thermo Fisher Scientific) and Agilent Nano Chips Bioanalyzer (Agilent Technologies). Depletion of ribosomal RNA was performed on 2–5 μg of total RNA using the Ribo-Zero rRNA Removal Kit (Illumina), and libraries were produced from 10–100 ng of ribosomal-depleted RNA using the NextFlex Rapid Directional RNA-Seq Kit (Bio Scientific) with 12 cycles of PCR amplification. Libraries were pooled in equimolar quantities and sequenced on the HiSeq4000 platform (Illumina) at Cancer Research UK (CRUK). Library preparation was performed by the W-MRC CSCI genomics facility. Reads were aligned to mouse genome reference GRCm38/mm10 with TopHat2 (Kim et al., 2013) v.2.1.0 (https://ccb.jhu.edu/software/tophat) using default parameters for paired end reads. Gene-wise counts were generated using featureCounts (Liao et al., 2014) based on annotation from the Ensembl GRCm38.86 release. Transcript counts were TMM normalized, and differentially expressed genes were called using a non-parametric algorithm specifically intended for use with data lacking replicates, with q > 0.9 using NOISeq-sim from NOISeq (Tarazona et al., 2011, 2015) v.2.30.0. For all NOISeq analyses, the following parameters were used: pnr = 0.2, nss = 5, v = 0.02; and seed “321” was used for each analysis.

### Western blot

Cells were lysed in RIPA buffer (Sigma) containing Complete-ULTRA protease-inhibitor and PhosSTOP phosphatase-inhibitor cocktails (Roche), and sonicated with a Bioruptor 200 (Diagenode) at high frequency, alternating 30 s on/off for 3 min. SDS-PAGE was performed using Bolt 10% Bis-Tris Plus gels (Thermo Fisher) in a Novex Mini-Cell (Thermo Fisher). Protein transfer was performed using the semi-dry iBlot2 system (Thermo Fisher) and iBlot Transfer Stacks (Thermo Fisher). Detection was achieved using horseradish peroxidase-linked secondary antibodies diluted 1:10,000 against the appropriate species (GE Healthcare) and the ECL Plus Western Blotting Detection System (GE Healthcare). See the supplemental experimental procedures for primary antibodies.

### Protein half-life analysis

Cells were treated with cycloheximide with or without addition of IAA and harvested at hourly intervals. Western blot analysis of Nanog protein levels was performed, and a relative standard curve was included using a 1 in 2 dilution series with four points from the uninduced sample. Western blots were quantitated using Image Studio Lite quantification software.

### ChIP-qPCR and ChIP-seq

ChIP was performed as previously described (Radzisheuskaya et al., 2013). For detailed protocols see the supplemental experimental procedures. Chromatin was analyzed by qPCR using a StepOnePlus real-time PCR system with recommended settings and Fast SYBR Green Master Mix. In addition, next-generation sequencing libraries were prepared using the ThruPLEX DNA-Seq Kit. Libraries were pooled in equimolar quantities and sequenced on the HiSeq4000 platform (Illumina) at CRUK. Library preparation was performed by the W-MRC CSCI genomics facility. Reads were aligned to mouse genome reference GRCm38/mm10 using Bowtie 2 (Langmead and Salzberg, 2012) v.2.3.4.3 with default parameters and deduplicated using SAMtools rmdup. For details of next-generation sequencing analysis, see the supplemental experimental procedures.

### ChIP-western blot

ChIP was performed as above, except that a single 40 min elution was performed. Instead of overnight reverse-cross-linking, sample loading buffer and reducing agent were added to the eluate, and samples were boiled for 40 min to denature protein and reverse cross links. Western blot was then performed as described above.

### Data availability

The accession number for all sequencing data reported in this paper is GEO: GSE168579.

## Author contributions

L.E.B. and J.C.R.S. conceived the study. L.E.B. designed and performed experiments and bioinformatics analysis, analyzed the data, supervised the study, and wrote the manuscript. M.R.P.A. performed experiments. J.C.R.S. supervised the study and wrote and approved the manuscript.

## Supplemental information

Document S1. Supplemental experimental procedures and Figures S1–S5

Document S2. Article plus supplemental information
