# Supplementary material for: Auxin-degron system identifies immediate mechanisms of OCT4
Source: Stem Cell Reports. 2021 Jun 17;16(7):1818–31. doi: 10.1016/j.stemcr.2021.05.016 (PMC8282470; doi:10.1016/j.stemcr.2021.05.016)
Supplement: Document S1. Supplemental experimental procedures and Figures S1–S5 [file mmc1.pdf]

Stem Cell Reports, Volume *16*

Supplemental Information

Auxin-degron system identifies immediate mechanisms of OCT4

Lawrence E. Bates, Mariana R.P. Alves, and José C.R. Silva

Figure S1. Characterization of cell lines. Relating to Figure 1.

A) Cells before and after conventional tamoxifen-induced CreER driven genetic ablation of *Pou5f1*. Uninduced cells grow as compact, round, domed colonies. 4 days after application of tamoxifen, cells have attained an enlarged, flattened morphology. Scalebar – 100µm. B) Oct4$^{-/-}$ Tet-OFF Oct4 ESCs (left) and O4AID ESCs share similar morphology, with mostly compact, round, domed colonies. Scalebar – 50µm. C) Oct4$^{-/-}$ NSCs (left) were reprogrammed in the presence of either wild-type OCT4 or OCT4-AID fusion protein. In both cases, similar round, domed colonies were formed (right). Scalebar – 100µm. D) RT-qPCR analysis of pluripotency and lineage markers following monolayer differentiation (N2B27) and suspension differentiation (embryoid body) of O4AID iPS and Oct4 iPSCs. RT-qPCR data represent the mean ± SD of three technical replicates.

Figure S1

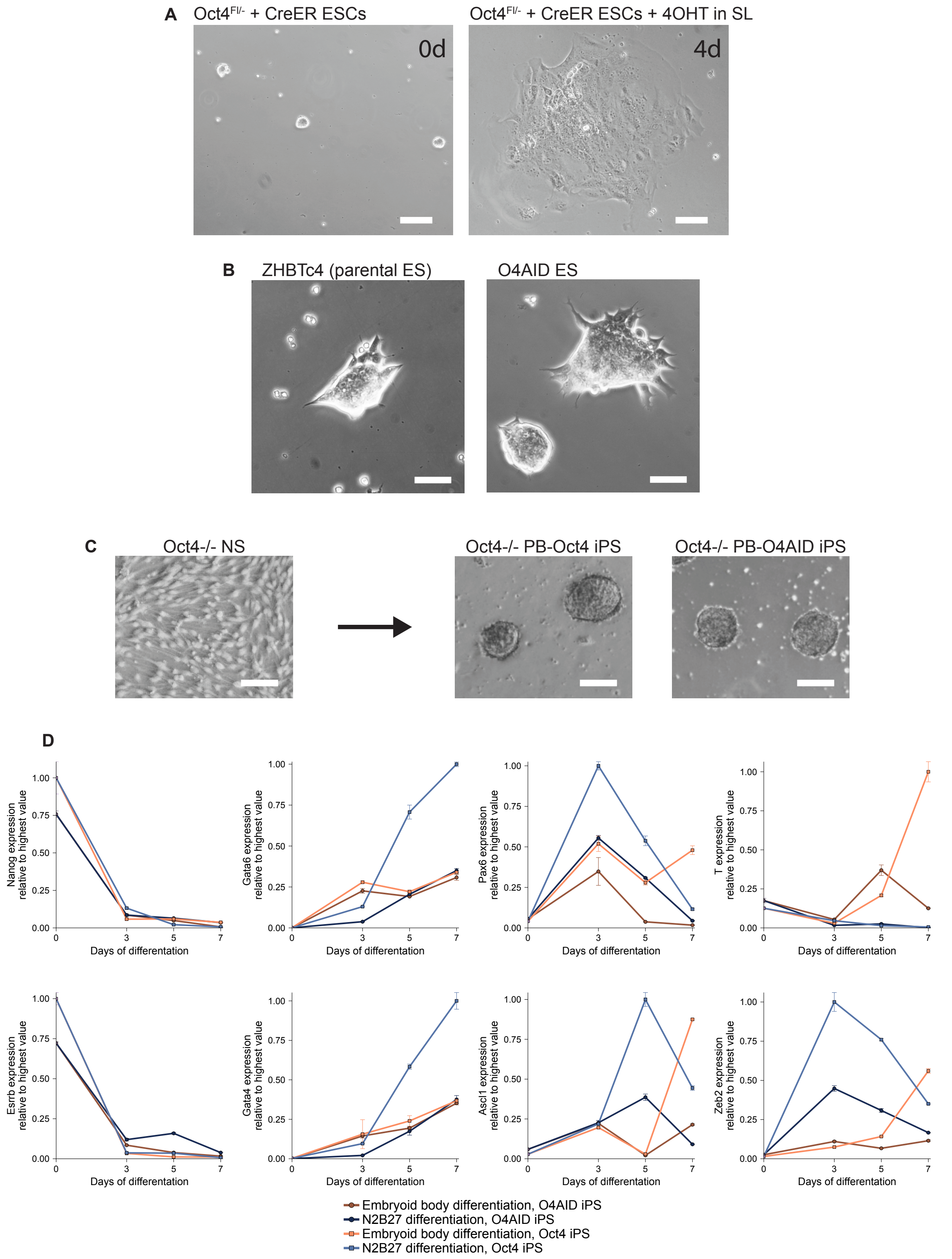

A
Oct4Fl/- + CreER ESCs
Oct4Fl/- + CreER ESCs + 4OHT in SL
0d
4d
B
ZHBTc4 (parental ES)
O4AID ES
C
Oct4-/- NS
Oct4-/- PB-Oct4 iPS
Oct4-/- PB-O4AID iPS
D
Nanog expression relative to highest value
Gata6 expression relative to highest value
Pax6 expression relative to highest value
T expression relative to highest value
Esrrb expression relative to highest value
Gata4 expression relative to highest value
Ascl1 expression relative to highest value
Zeb2 expression relative to highest value
Days of differentiation
Embryoid body differentiation, O4AID iPS
N2B27 differentiation, O4AID iPS
Embryoid body differentiation, Oct4 iPS
N2B27 differentiation, Oct4 iPS

Figure S2. Expression changes following induction. Relating to Figure 2.

RT-qPCR analysis of pluripotency (top) and trophectoderm (bottom) associated genes in $Oct4^{F/-}$ CreER ESCs (A), eGFPAID ESCs (B),O4AID ESCs (C), and O4AID iPSCs (D), in a timecourse following induced depletion of Oct4 or eGFP with 4OHT or IAA. RT-qPCR data represent the mean ± SD of three technical replicates.

Figure S2

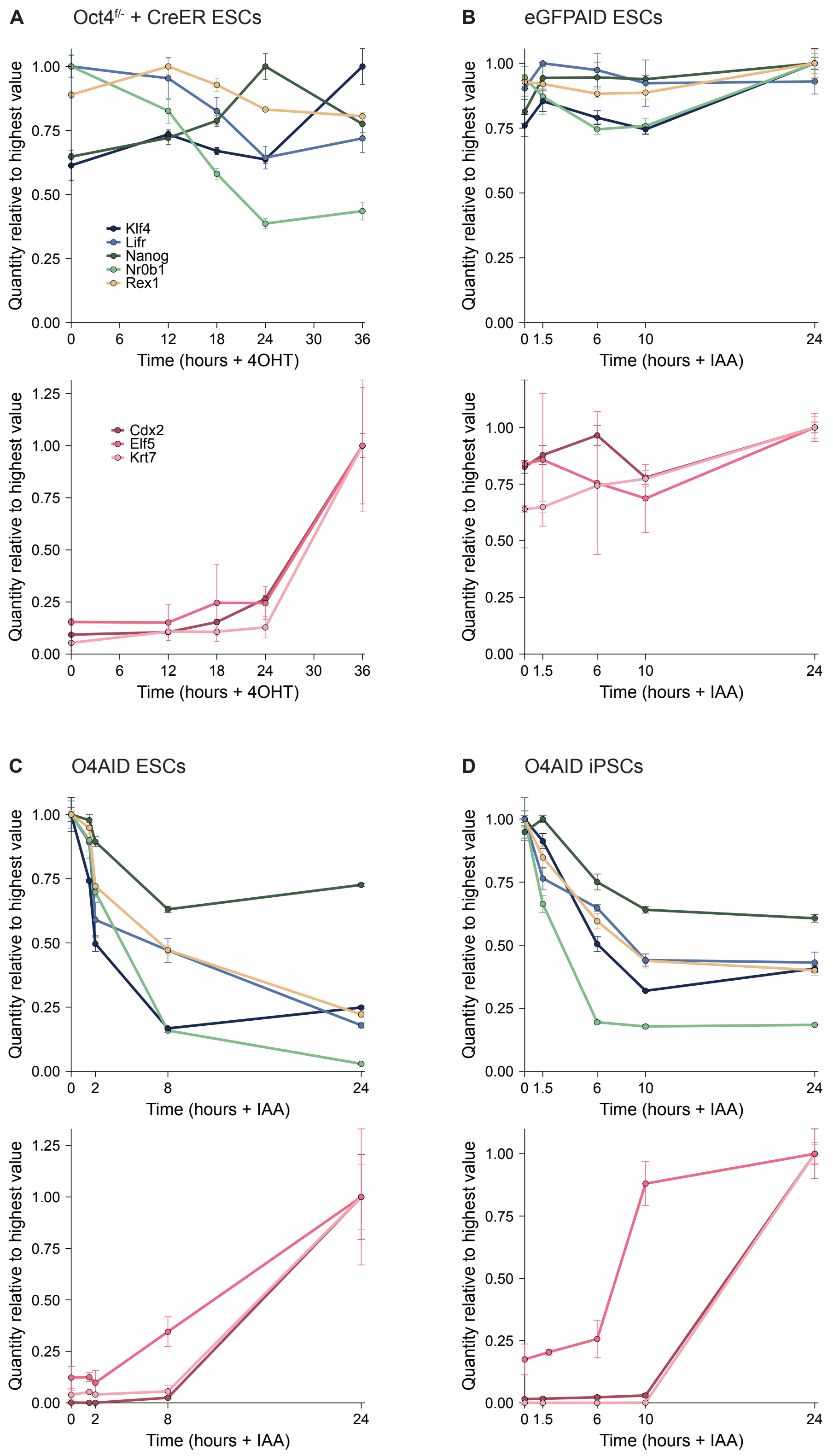
A
Oct4f/- + CreER ESCs
B
eGFPAID ESCs
C
O4AID ESCs
D
O4AID iPSCs
Quantity relative to highest value
Klf4
Lifr
Nanog
Nr0b1
Rex1
Cdx2
Elf5
Krt7
Time (hours + 4OHT)
Time (hours + IAA)

Figure S3. H3K27ac ChIP analysis following induction. Relating to Figure 3.

A) ChIP-seq analysis of H3K27ac at the *Tet1* enhancer (top) and the *Tet2* enhancer (bottom) in O4AID ESCs before and 1.5 hours after addition of IAA. Genomic coordinates refer to the GRCm38/mm10 genome assembly, and gene intron/exon annotations are taken from Ensembl. OCT4 binding sites generated from ChIP-seq data from Marson et al. 2008 indicated in purple. B&C) ChIP-qPCR analysis of H3K27ac at B) the *Klf4* distal enhancer and C) the *Prdm14* distal enhancer in O4AID ESCs (left), O4AID iPSCs (right) and eGFPAID ESCs (bottom) before and 1.5 hours after addition of IAA. ChIP-qPCR data represent the mean ± SD of three IPs for H3K27ac, and the mean of three technical replicates of a single IP for IgG. ND = not detected. D) Violin and box plot showing H3K27ac signal before and 1.5 hours after addition of IAA at all detected H3K27ac peaks. Peaks with signal below the indicated threshold both before and after induction were consider to be background and were excluded from further analysis. E) Violin plot showing log2-fold change in H3K27ac signal between uninduced and 1.5 hour IAA treated O4AID ESCs at the peaks below the threshold indicated in D).

Figure S3

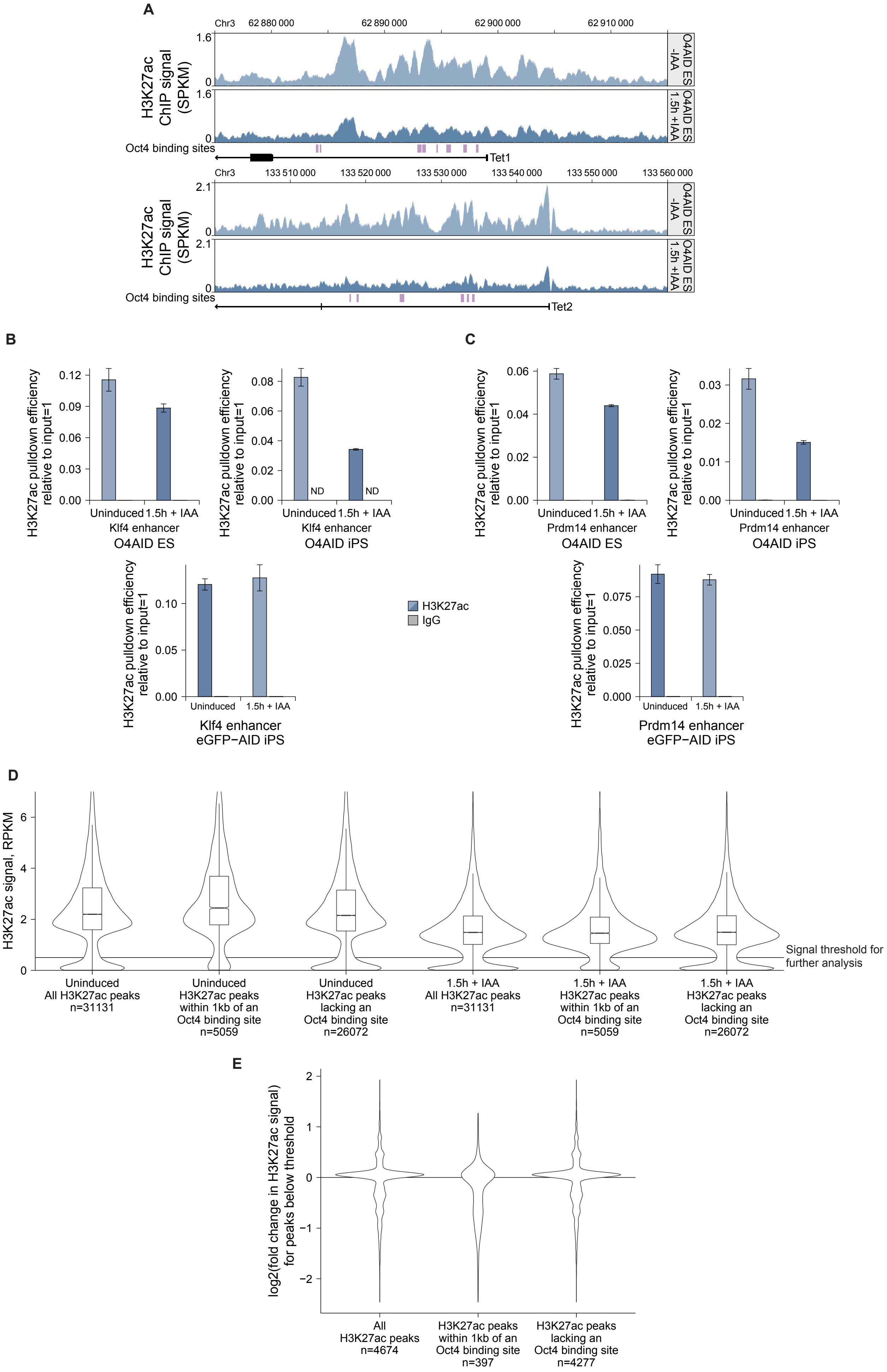

A
Chr3
62 880 000
62 890 000
62 900 000
62 910 000
H3K27ac ChIP signal (SPKM)
1.6
0
O4AID ES -IAA
O4AID ES 1.5h +IAA
Oct4 binding sites
Tet1
133 510 000
133 520 000
133 530 000
133 540 000
133 550 000
133 560 000
2.1
Tet2
B
H3K27ac pulldown efficiency relative to input=1
Uninduced
1.5h + IAA
Klf4 enhancer
O4AID ES
ND
O4AID iPS
Klf4 enhancer eGFP−AID iPS
C
Prdm14 enhancer
O4AID ES
O4AID iPS
Prdm14 enhancer eGFP−AID iPS
H3K27ac
IgG
D
H3K27ac signal, RPKM
Signal threshold for further analysis
Uninduced All H3K27ac peaks n=31131
Uninduced H3K27ac peaks within 1kb of an Oct4 binding site n=5059
Uninduced H3K27ac peaks lacking an Oct4 binding site n=26072
1.5h + IAA All H3K27ac peaks n=31131
1.5h + IAA H3K27ac peaks within 1kb of an Oct4 binding site n=5059
1.5h + IAA H3K27ac peaks lacking an Oct4 binding site n=26072
E
log2(fold change in H3K27ac signal) for peaks below threshold
All H3K27ac peaks n=4674
H3K27ac peaks within 1kb of an Oct4 binding site n=397
H3K27ac peaks lacking an Oct4 binding site n=4277

Figure S4. Analysis of OCT4 and NANOG following induction. Relating to Figure 4.

A) ChIP-Western for OCT4AID in O4AID ESCs, either uninduced or following 1.5 hours of treatment with IAA. Input samples were taken from sheared, crosslinked chromatin prior to immunoprecipitation. Input was diluted so that one tenth the amount was loaded compared to IP samples. Note, a high molecular weight band in the uninduced pull-down lane likely represents OCT4 still bound to chromatin due to incomplete reverse crosslinking. Non-neighbouring lanes from the same blot have been aligned for comparison (full blot in B)). B) Complete blot that lanes in A) are taken from. C-D) Analysis of NANOG binding to pluripotent regulatory sequences in O4AID iPSCs. C) Protein level of OCT4 and NANOG (Western blot) in O4AID iPSCs before and 2 hours after addition of IAA with α-TUBULIN as a loading control. D) ChIP qPCR following pulldown of NANOG or using normal IgG negative control at NANOG binding sites or a negative control locus in O4AID iPSCs. NANOG pulldown represents mean ± SD of three IPs; IgG pulldown represents the mean of three technical replicates of a single IP.

Figure S4

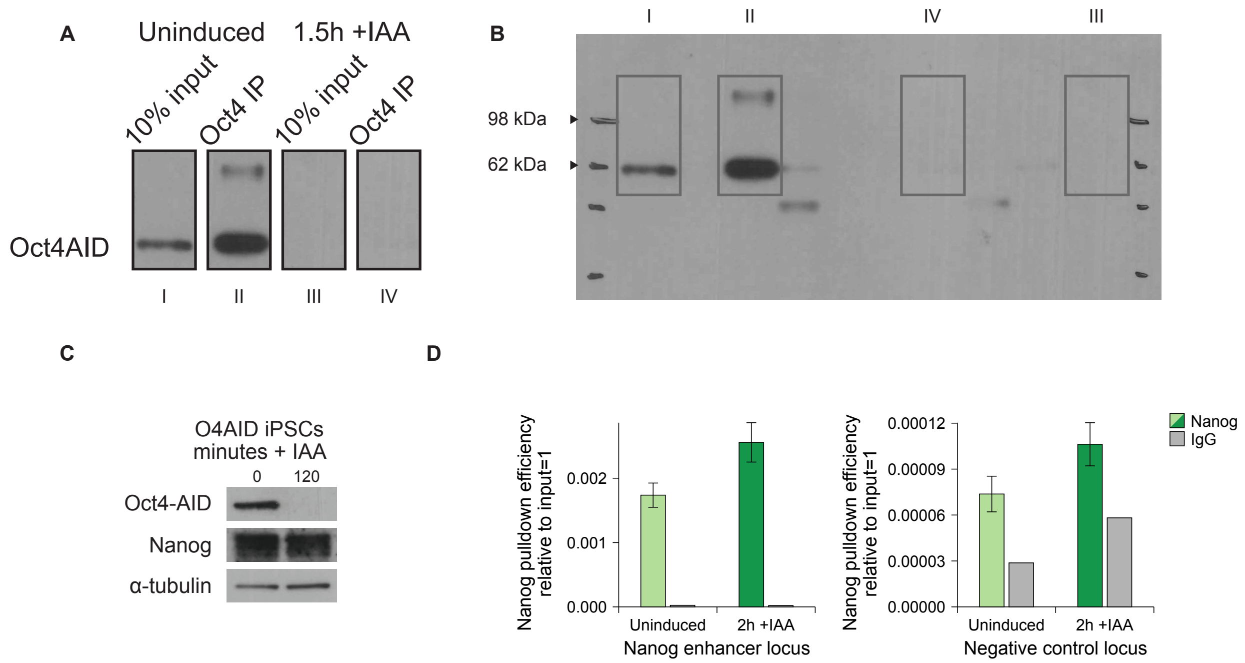

A
Uninduced
1.5h +IAA
10% input
Oct4 IP
10% input
Oct4 IP
Oct4AID
I
II
III
IV
B
I
II
IV
III
98 kDa
62 kDa
C
O4AID iPSCs
minutes + IAA
0
120
Oct4-AID
Nanog
α-tubulin
D
Nanog pulldown efficiency relative to input=1
0.002
0.001
0.000
Uninduced
2h +IAA
Nanog enhancer locus
Nanog pulldown efficiency relative to input=1
0.00012
0.00009
0.00006
0.00003
0.00000
Uninduced
2h +IAA
Negative control locus
Nanog
IgG

Figure S5. Analysis of NANOG stability and global chromatin binding. Relating to Figure 5.

A&B) Analysis of NANOG protein half-life in O4AID ES with and without OCT4 depletion. A) Western blot analysis of NANOG protein level in a timecourse following cycloheximide treatment, with or without addition of IAA. Standard curve is equivalent to 1X 0h uninduced sample, 0.5X, 0.25X, 0.125X. B) Plot of natural log of NANOG protein quantity against time used to calculate the decay constant, λ, from the equation $\ln(x(t)) = \ln(x_0) + \lambda t$. From this, the protein half-life is calculated as $t_{1/2} = \ln(2)/\lambda$. C) Violin and box plot showing NANOG signal before and 1.5 hours after addition of IAA at all detected NANOG peaks. Peaks with signal below the indicated threshold both before and after induction were consider to be background and were excluded from further analysis. D) Violin plot showing log2-fold change in NANOG signal between uninduced and 1.5 hour IAA treated O4AID ESCs at the peaks below the threshold indicated in C).

Figure S5

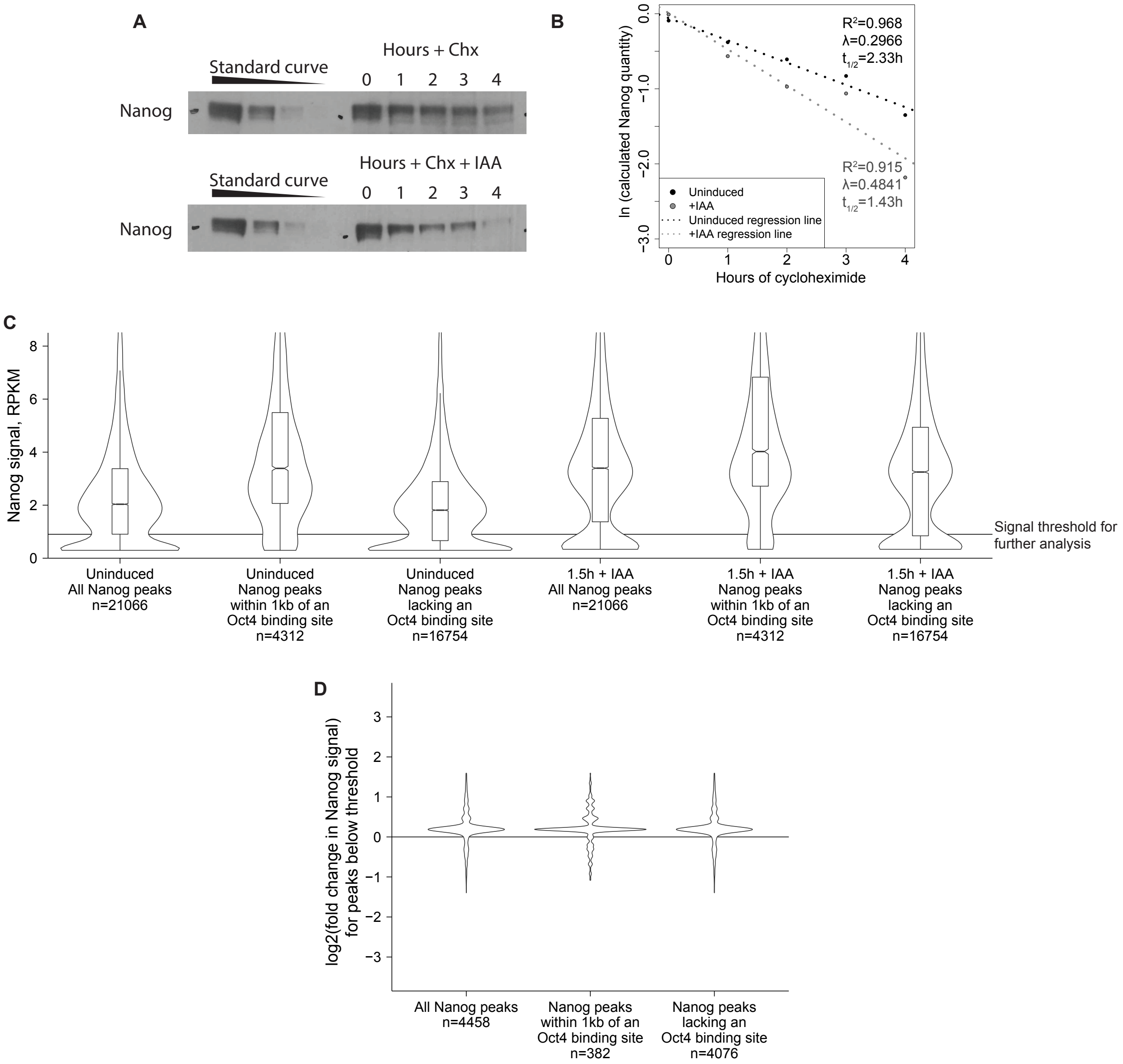

A
Hours + Chx
Standard curve
0 1 2 3 4
Nanog
Hours + Chx + IAA
Standard curve
0 1 2 3 4
Nanog
B
R²=0.968
λ=0.2966
t1/2=2.33h
R²=0.915
λ=0.4841
t1/2=1.43h
Uninduced
+IAA
Uninduced regression line
+IAA regression line
ln (calculated Nanog quantity)
Hours of cycloheximide
C
Nanog signal, RPKM
Signal threshold for further analysis
Uninduced All Nanog peaks n=21066
Uninduced Nanog peaks within 1kb of an Oct4 binding site n=4312
Uninduced Nanog peaks lacking an Oct4 binding site n=16754
1.5h + IAA All Nanog peaks n=21066
1.5h + IAA Nanog peaks within 1kb of an Oct4 binding site n=4312
1.5h + IAA Nanog peaks lacking an Oct4 binding site n=16754
D
log2(fold change in Nanog signal) for peaks below threshold
All Nanog peaks n=4458
Nanog peaks within 1kb of an Oct4 binding site n=382
Nanog peaks lacking an Oct4 binding site n=4076

Supplemental Experimental Procedures

Chromatin immunoprecipitation

Briefly, 10×10^6 cells per IP for transcription factors or 5×10^6 cells per IP for histone modifications were fixed for 10 min in 1% formaldehyde. Fixation was halted by addition of an excess of glycine. Cells were washed with ice-cold PBS. Nuclei were isolated: Cells were incubation with Lysis Buffer 1 (50 mM HEPES pH 7.5, 140 mM NaCl,1 mM EDTA, 10% Glycerol, 0.5% NP40, 0.25% Tx100) at 4 °C for 10 mins, pelleted, then incubated with Lysis Buffer 2 (10 mM Tris pH 8.0, 200 mM NaCl, 1 mM EDTA, 0.5 mM EGTA) at 4 °C for 10 mins. Nuclei were pelleted then resuspended in Shearing Buffer (50 mM Tris pH 8.0, 1% SDS, 10 mM EDTA) and sonicated to obtain an average DNA fragment size of 500 base pairs. Lysates were diluted 1:10 in dilution buffer (50 mM Tris–HCl at pH 8.0, 167 mM NaCl, 1.1% Triton X-100 and 0.11% sodium deoxycholate) and pre-cleared for 2 h at 4 °C with Dynabeads Protein G magnetic beads (Invitrogen) that were pre-incubated with isotypic IgG antibody. Supernatent was collected, a 10% input sample was taken for relative quantitation, and the chromatin was the incubated overnight at 4 °C with 2 µg of antibody or 2 µg of isotypic IgG control. Lysates were then incubated for 1 h at 4 °C with BSA-blocked Dynabeads Protein G magnetic beads, and the beads were washed twice in wash buffer 1 (50 mM Tris pH 8.0, 0.1% SDS, 0.1% Sodium deoxycholate, 1% Tx100, 150 mM NaCl, 1 mM EDTA, 0.5 mM EGTA), once in Wash Buffer 2 (50 mM Tris pH 8.0, 0.1% SDS, 0.1% sodium deoxycholate, 1% Tx100, 500 mM NaCl, 1 mM EDTA, 0.5 mM EGTA), once in Wash Buffer 3 (50 mM Tris pH 8.0, 250 mM LiCl, 0.5% Sodium deoxycholate, 0.5% NP40, 1 mM EDTA, 0.5 mM EGTA) and twice in wash buffer 4 (50 mM Tris pH 8.0, 10 mM EDTA and 5 mM EGTA). Chromatin was eluted twice for a total of 30 min at 65 °C in elution buffer (1% SDS and 0.1 M $NaHCO_3$). NaCl was added to samples and inputs to a final concentration of 200 mM, before incubating overnight at 65 °C to reverse the crosslinking and DNA was purified using the QIAquick PCR Purification kit (Qiagen).

ChIP-seq analysis

Normalized enrichment tracks were generated from aligned reads with the MACS2 (Liu, 2014) callpeak function using the –SPMR flag. H3K27ac peaks were called using MACS2 callpeak function on pooled mapped reads of 3 independent IPs from uninduced samples. For analysis of H3K27ac signal intensity, mapped reads from 3 independent IPs were pooled and RPKM normalized using DiffBind (Stark and Brown, 2017). Peaks with RPKM < 0.5 in both uninduced and induced samples were considered to be background and excluded from further analysis. Subsets of peaks located within 1kb of Oct4 peaks were determined by analysis of published ChIP-seq data; reads were deduplicated and aligned as above, and peaks were called using MACS2 callpeak function with whole cell extract used as control samples.

Nanog peaks were called from aligned reads using MACS2 callpeak function on pooled ChIP-seq data from two independent IPs each from both uninduced and IAA treated samples. For analysis of Nanog signal at binding sites, mapped reads from two independent IPs each of treated and untreated samples were pooled and RPKM normalized using deepTools2 (Ramírez et al., 2016) bamCoverage and blacklisted regions from the ENCODE project consortium were excluded, then a heatmap and summary plot were generated using deepTools2 computeMatrix and plotHeatmap tools. For pairwise analysis of Nanog binding at peaks called from pooled data from both uninduced and IAA treated samples, centred on the peak summit and extending a total of 350bp, replicates were pooled and RPKM normalized using DiffBind. Peaks with an RPKM < 0.9 in both uninduced and induced samples were considered to be background and excluded from further analysis. Subsets of peaks corresponding to active promoters, active enhancers and repressed regions were assigned using published ChIP-seq peaksets from ENSEMBL, using the following criteria: active promoters, within 1kb of PolII and either overlapping H3K4me1 or H3K4me3; active enhancer, within 1kb of P300 and overlapping H3K27ac; repressed region, overlapping either H3K9me3 or H3K27me3.

Public datasets used

Dataset	Source	Analysis
Bruce4 ES EP300 ChIP-seq peakset	Ensembl (release 100)	SWEmbl

Bruce4 ES PolII ChIP-seq peakset	Ensembl (release 100)	SWEmbl
Bruce4 ES H3K27ac ChIP-seq peakset	Ensembl (release 100)	SWEmbl
Bruce4 ES H3K27me3 ChIP-seq peakset	Ensembl (release 100)	CCAT
Bruce4 ES H3K4me1 ChIP-seq peakset	Ensembl (release 100)	CCAT
Bruce4 ES H3K4me3 ChIP-seq peakset	Ensembl (release 100)	SWEmbl
Bruce4 ES H3K9me3 ChIP-seq peakset	Ensembl (release 100)	CCAT
V6.5 ES Oct4 ChIP-seq data	Marson et al. 2008, GEO accession GSM307137	See above
V6.5 ES whole cell extract data	Marson et al. 2008, GEO accessions GSM307154 and GSM307155	See above

Antibodies

Target	Source	Catalogue number
Polyclonal goat anti-Oct4	Santa Cruz Biotechnology	sc-8628
Monoclonal mouse anti-alpha-Tubulin	Abcam	ab7291
Polyclonal rabbit anti-H3K27ac	Abcam	ab4729
Monoclonal rat anti-Nanog	eBioscience	14-5761-80
Monoclonal rat anti-Sox2	eBioscience	14-9811-80

qPCR primers and probesets

Target	Use	Source	Catalogue number / sequence
Oct4	RT-qPCR	ThermoFisher Scientific	Mm00658129_gH
Nanog	RT-qPCR	ThermoFisher Scientific	Mm02384862_g1
Rex1	RT-qPCR	ThermoFisher Scientific	Mm03053975_g1
Klf4	RT-qPCR	ThermoFisher Scientific	Mm00516104_m1
Lifr	RT-qPCR	ThermoFisher Scientific	Mm00442942_m1
Nr0b1	RT-qPCR	ThermoFisher Scientific	Mm00431729_m1
Cdx2	RT-qPCR	ThermoFisher Scientific	Mm01212280_m1
Elf5	RT-qPCR	ThermoFisher Scientific	Mm00468732_m1
Krt7	RT-qPCR	Sigma Aldrich	MMUS_NM_033073_1
Gata6	RT-qPCR	ThermoFisher Scientific	Mm00802636_m1
Pax6	RT-qPCR	ThermoFisher Scientific	Mm00443081_m1
T	RT-qPCR	ThermoFisher Scientific	Mm01318252_m1
Gata4	RT-qPCR	ThermoFisher Scientific	Mm00484689_m1
Ascl1	RT-qPCR	ThermoFisher Scientific	Mm03058063_m1
Zeb2	RT-qPCR	ThermoFisher Scientific	Mm00497193_m1
Gapdh	RT-qPCR	ThermoFisher Scientific	4352339E
Retroviral Oct4	RT-qPCR	Sigma Aldrich	F: TGGTACGGGAAATCACAAGTTTGTA<br>R: GGTGAGAAGGCGAAGTCTGAAG

			Probe: FAM-CACCTTCCCCATGGCTG-MGB
Retroviral cMyc	RT-qPCR	Sigma Aldrich	F: TGGTACGGGAAATCACAAGTTTGTA<br>R: GGTCATAGTTCCTGTTGGTGAAGTT<br>Probe: FAM-CCCTTCACCATGCCCC-MGB
Retroviral Klf4	RT-qPCR	Sigma Aldrich	F: TGGTACGGGAAATCACAAGTTTGTA<br>R: GAGCAGAGCGTCGCTGA<br>Probe: FAM-CCCCTTCACCATGGCTG-MGB
Klf4 enhancer	ChIP-qPCR	Sigma Aldrich	F: TGTCCTCTCCACTCCCACAA<br>R: AGGAGTGACTGCGTCAAACA
Prdm14 enhancer	ChIP-qPCR	Sigma Aldrich	F: CTACCCTAGACCCCACCTCC<br>R: GGGTCCCCTCTCAAAACACC
Nanog enhancer	ChIP-qPCR	Sigma Aldrich	F: CCCACCTGTCCCTAGTCCCCGCT<br>R: TTGGAACTAGCTGTGTGGGTGGGG
Oct4 distal enhancer	ChIP-qPCR	Sigma Aldrich	F: GCATAACAAAGGTGCATGATAGCT<br>R: AAATAAAGGCAGCGACTTGGAA
Chr6 negative control locus	ChIP-qPCR	Sigma Aldrich	F: ACTACCCAAACTATTGCTCCTGA<br>R: GCTTAACCTGCTCTCCCAGG

Supplemental References

Liu, T. (2014). Use model-based Analysis of ChIP-Seq (MACS) to analyze short reads generated by sequencing protein-DNA interactions in embryonic stem cells. Methods Mol. Biol. *1150*, 81–95.

Marson, A., Levine, S.S., Cole, M.F., Frampton, G.M., Brambrink, T., Johnstone, S., Guenther, M.G., Johnston, W.K., Wernig, M., Newman, J., et al. (2008). Connecting microRNA genes to the core transcriptional regulatory circuitry of embryonic stem cells. Cell *134*, 521–533.

Ramírez, F., Ryan, D.P., Grüning, B., Bhardwaj, V., Kilpert, F., Richter, A.S., Heyne, S., Dündar, F., and Manke, T. (2016). deepTools2: a next generation web server for deep-sequencing data analysis. Nucleic Acids Res. *44*, W160-5.

Stark, R., and Brown, G. (2017). DiffBind: differential binding analysis of ChIP-seq peak data. http://bioconductor.org/packages/release/bioc/vignettes/DiffBind/inst/doc/DiffBind.pdf
